# Supplementary material for: Bivalent antibody pliers inhibit β-tryptase by an allosteric mechanism dependent on the IgG hinge
Source: Nat Commun. 2020 Dec 22;11:6435. doi: 10.1038/s41467-020-20143-x (PMC7755903; doi:10.1038/s41467-020-20143-x)
Supplement: Supplementary file 1 — Supplementary Information [file 41467_2020_20143_MOESM1_ESM.pdf]

Supplementary Information for

Bivalent antibody pliers inhibit β-tryptase by an allosteric mechanism dependent on the IgG hinge

Henry R. Maun[1], Rajesh Vij[2], Benjamin T. Walters[3], Ashley Morando[3], Janet K. Jackman[4], Ping Wu[5], Alberto Estevez[5], Xiaocheng Chen[2], Yvonne Franke[6], Michael T. Lipari[1], Mark S. Dennis[2], Daniel Kirchhofer[1], Claudio Ciferri[5], Kelly M. Loyet[3], Tangsheng Yi[4], Charles Eigenbrot[5], Robert A. Lazarus[1,*], James T. Koerber[2,*]

*Correspondence to laz@gene.com (R.A.L.) or koerberj@gene.com (J.T.K.).

This PDF file includes:

Supplementary Table 1 and 2

Supplementary Figures 1-12

Supplementary Note 1

Other supplementary materials for this manuscript include the following:

Supplementary Data 1 and 2

Supplementary Information

Supplementary Table 1.
EM data collection, refinement and validation statistics

	E104.v1 Fab tryptase complex (EMDB-21389)
**Data collection and processing**	
Magnification	67000
Voltage (kV)	120
Electron exposure (e–/Å^2)	40 e–/Å^2
Defocus range (μm)	−0.8 to −1.3 μm.
Pixel size (Å)	2.2 Å
Symmetry imposed	D2
Initial particle images (no.)	12451
Final particle images (no.)	12451
Map resolution (Å)	15 Å
FSC threshold	0.5
Map resolution range (Å)	
**Refinement**	
Initial model used (PDB code)	Ab initio
Model resolution (Å)	15 Å
FSC threshold	0.5
Model resolution range (Å)	
Map sharpening *B* factor (Å^2)	
Model composition	
Non-hydrogen atoms	
Protein residues	
Ligands	
B factors (Å^2)	
Protein	
Ligand	
R.m.s. deviations	
Bond lengths (Å)	
Bond angles (°)	
Validation	
MolProbity score	
Clashscore	
Poor rotamers (%)	
Ramachandran plot	
Favored (%)	
Allowed (%)	
Disallowed (%)	

Supplementary Table 2. Amide protons of tryptase residues that showed slower hydrogen-deuterium exchange (HDX) when bound to the either E104.v1 or v2 Fabs as determined by overlapping peptides. For comparison, the contact residues that are within 4 Å of E104.v1 Fab in the X-ray structure are shown.

**Tryptase residues in contact with E104 Fabs**		
**E104.v1 (HDX)**	**E104.v2 (HDX)**	**Contact residues in crystal structure**
K26-W27	S25-W27	-
-	-	W38
-	F41-G43	-
I47-Q50	-	Q50
T54-A55	-	-
-	-	-
D60e-L61	D60e-L61	D60e-A62
V66-L68	V66-E70	R65
-	Q81-L83	Q81-R87
V85-I88	I88	-
L108-E110	L108-E110	E107-E110
H119-T122	-	-
-	V160-I162	-
D179	-	-
R230-V231	-	-
-	-	-
K244-K245	-	-

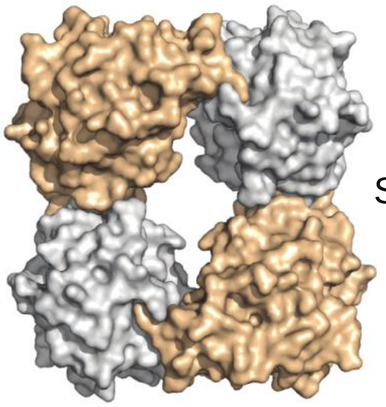

Supplementary Figure 1. Structure of tryptase tetramer. Protomers A and C (white) and protomers B and D (beige) assemble into tetrameric tryptase (PDB accession 1A0L). The complex is stabilized via two copies of a large interface (protomer A-D and B-C) and two copies of a small interface (protomer A-B and C-D).

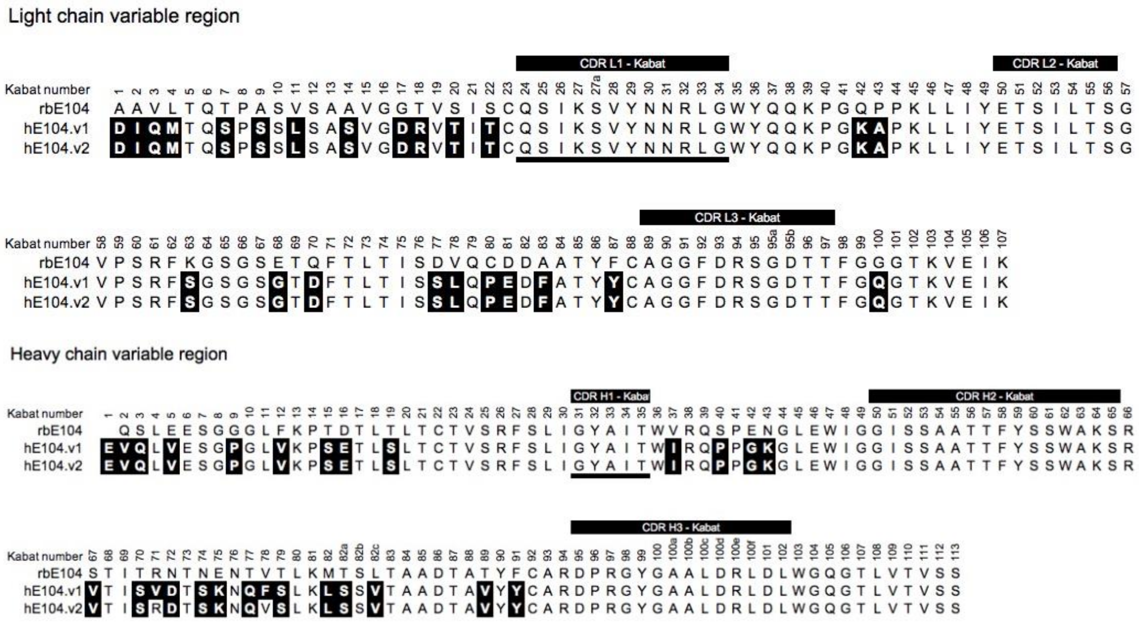

Supplementary Figure 2. Sequence alignment of the parental rabbit E104 and humanized variants (hE104.v1 and hE104.v2).

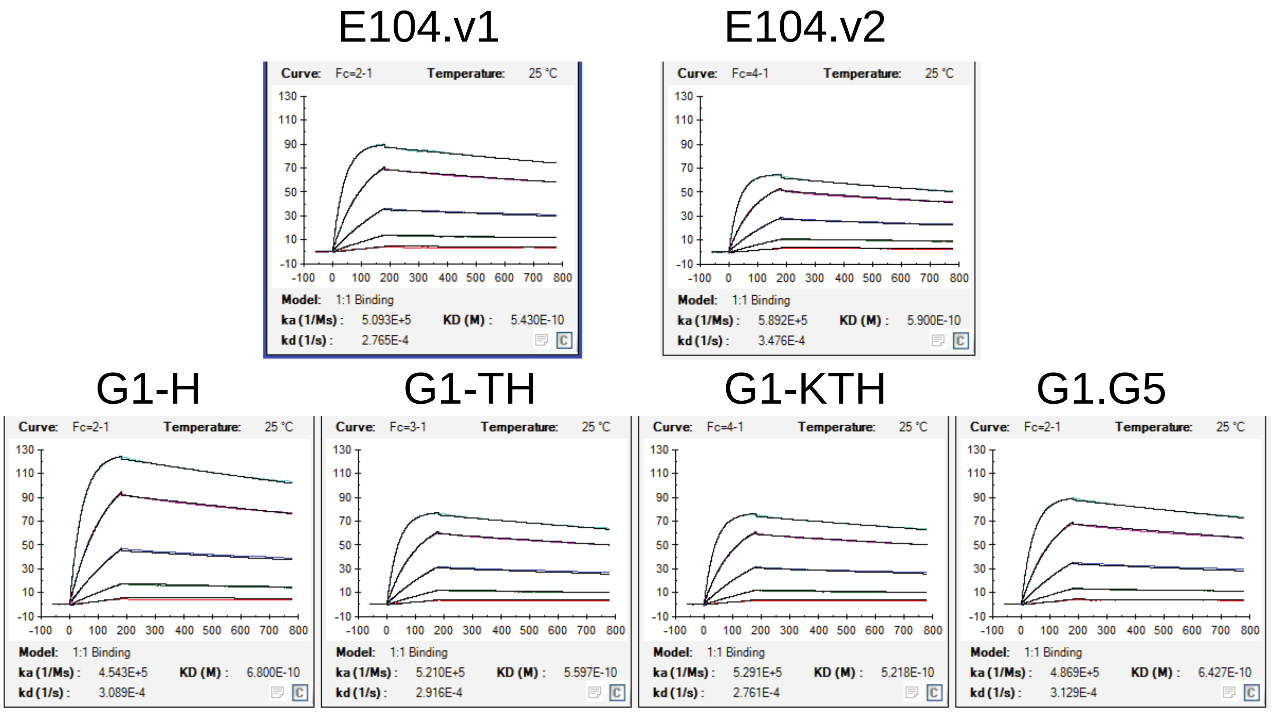

Supplementary Figure 3. Representative Biacore sensograms of E104.v1, E104.v2, and a subset of E104.v2 hinge variants.

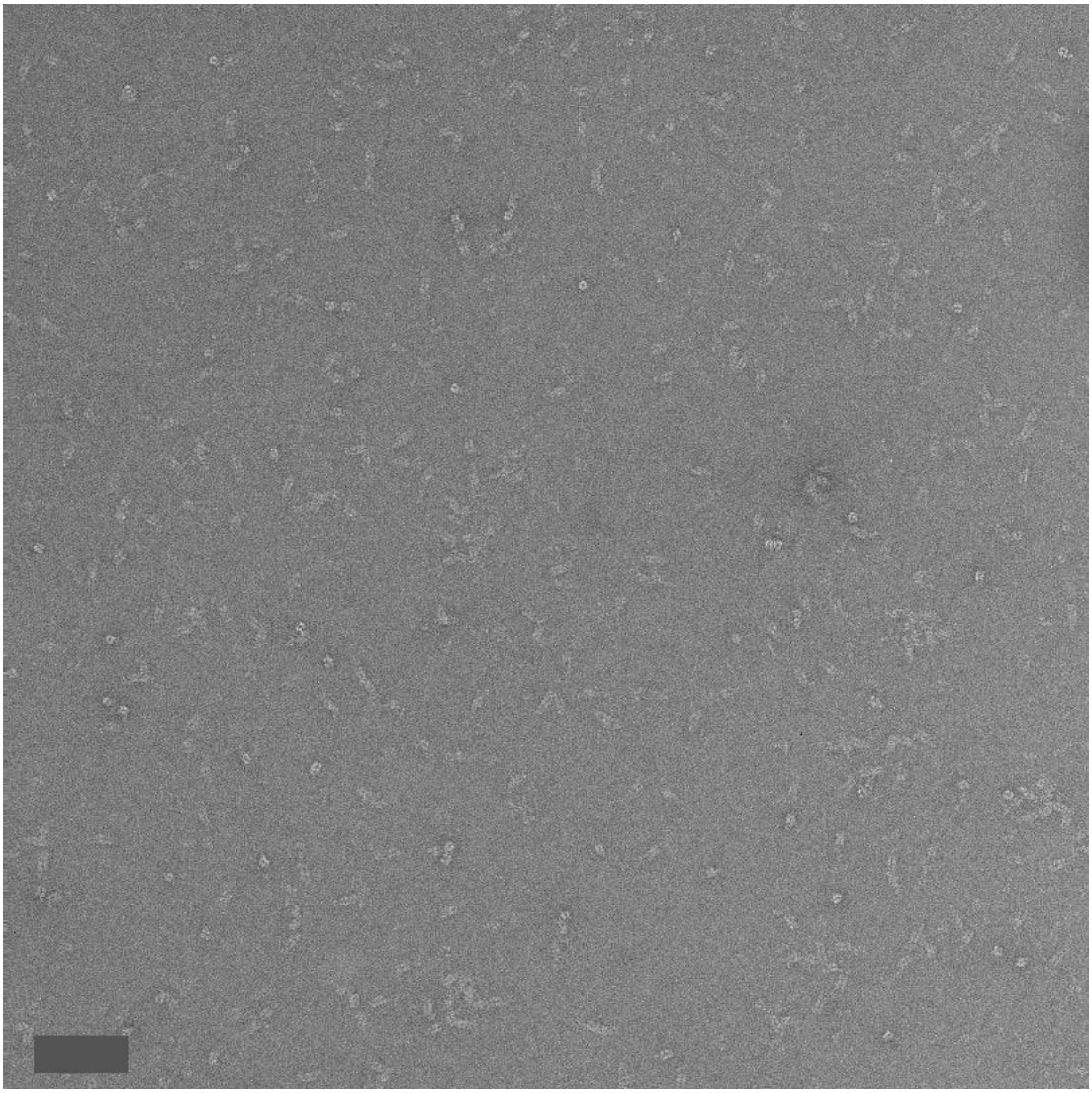

Supplementary Figure 4. Representative EM image with one experiment showing individual particles of βI-tryptase in complex with E104.v1 Fab. Scale bar denotes 80 nm.

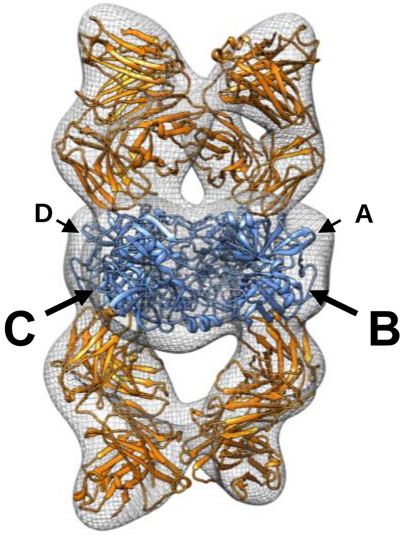

Supplementary Figure 5. 3D reconstruction of tetrameric βI-tryptase in complex with Fab E104.v1. Docking tryptase tetramer (blue) and Fab E104.v1 (orange) into the EM density maps at 15 Å resolution shows a 4:4 protomer:Fab stochiometric architecture.

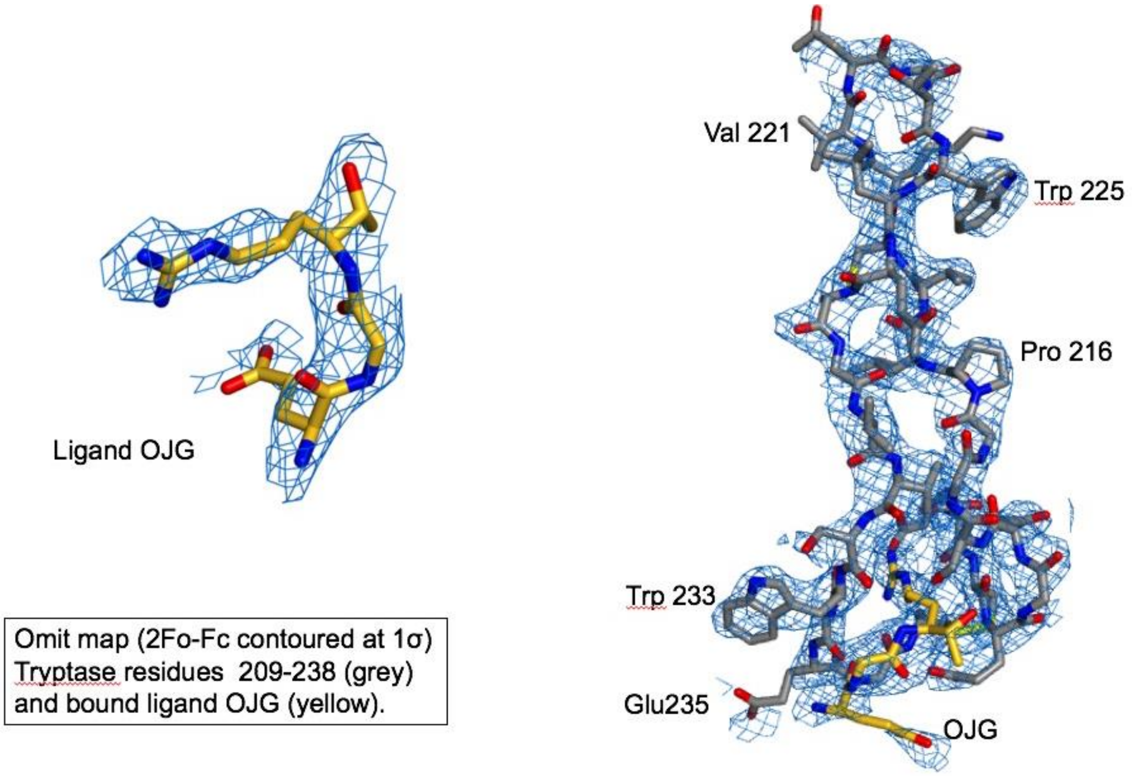

Supplementary Figure 6. Representative electron density map from βI-tryptase:Fab E104.v1.

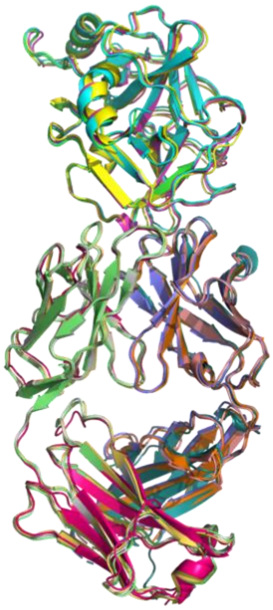

Supplementary Figure 7. Comparison of βI-tryptase:Fab structures within the 4:4 protomer:Fab complex. A structural overlay of each of the four Fabs and tryptase protomers observed in X-ray structure shows a high degree of similarity with main chain rmsds ranging from 0.85-1.44 Å.

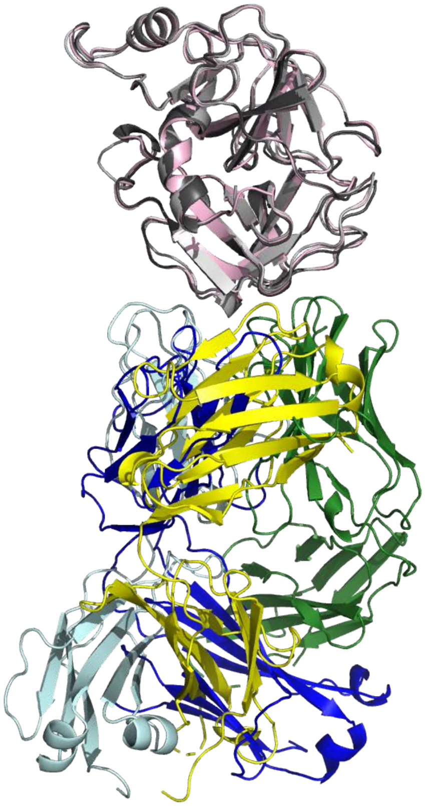

Supplementary Figure 8. Comparison of tryptase structures. Structural overlay of tryptase bound to E104.v1 Fab (PDB accession 6VVU; LC and HC are shown in dark blue and green, respectively, and tryptase is shown in light grey), 31A.v11 Fab (PDB accession 6O1F; LC and HC are shown in light blue and yellow, respectively, and tryptase is shown in dark grey), or in the absence of an antibody (PDB accession 1A0L; tryptase is shown in pink) shows high degree of similarity in tryptase conformations.

Supplementary Figure 9. HDX peptides of βI-tryptase in complex with E104.v1 Fab

+E104v1

Dashed black line is theoretical unprotected uptake trace. Solid black line is βI-tryptase alone. Solid red line is βI-tryptase in complex with E104.v1 Fab. Samples were analyzed in duplicate.

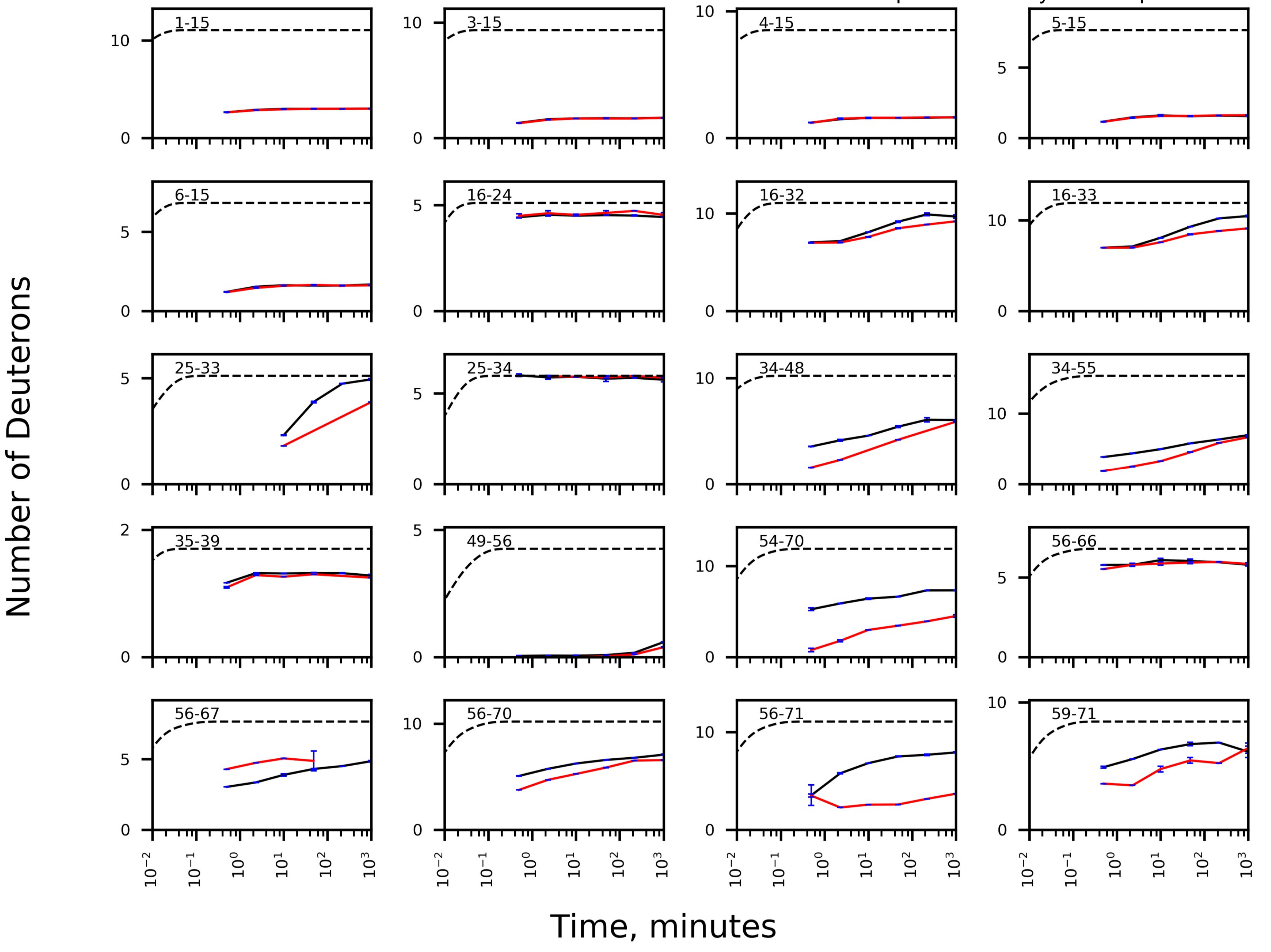

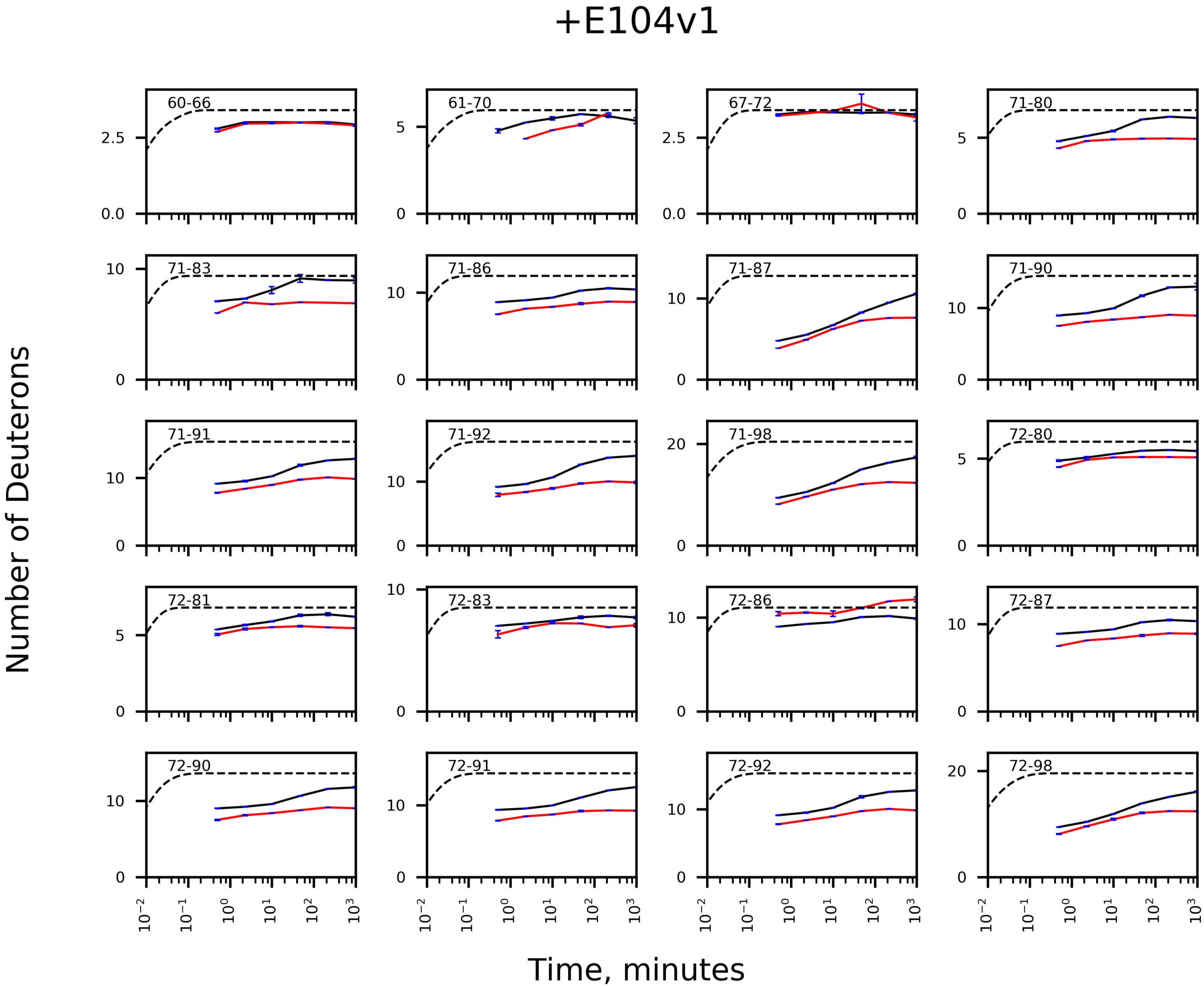

+E104v1
Number of Deuterons
Time, minutes
60-66
61-70
67-72
71-80
71-83
71-86
71-87
71-90
71-91
71-92
71-98
72-80
72-81
72-83
72-86
72-87
72-90
72-91
72-92
72-98

+E104v1

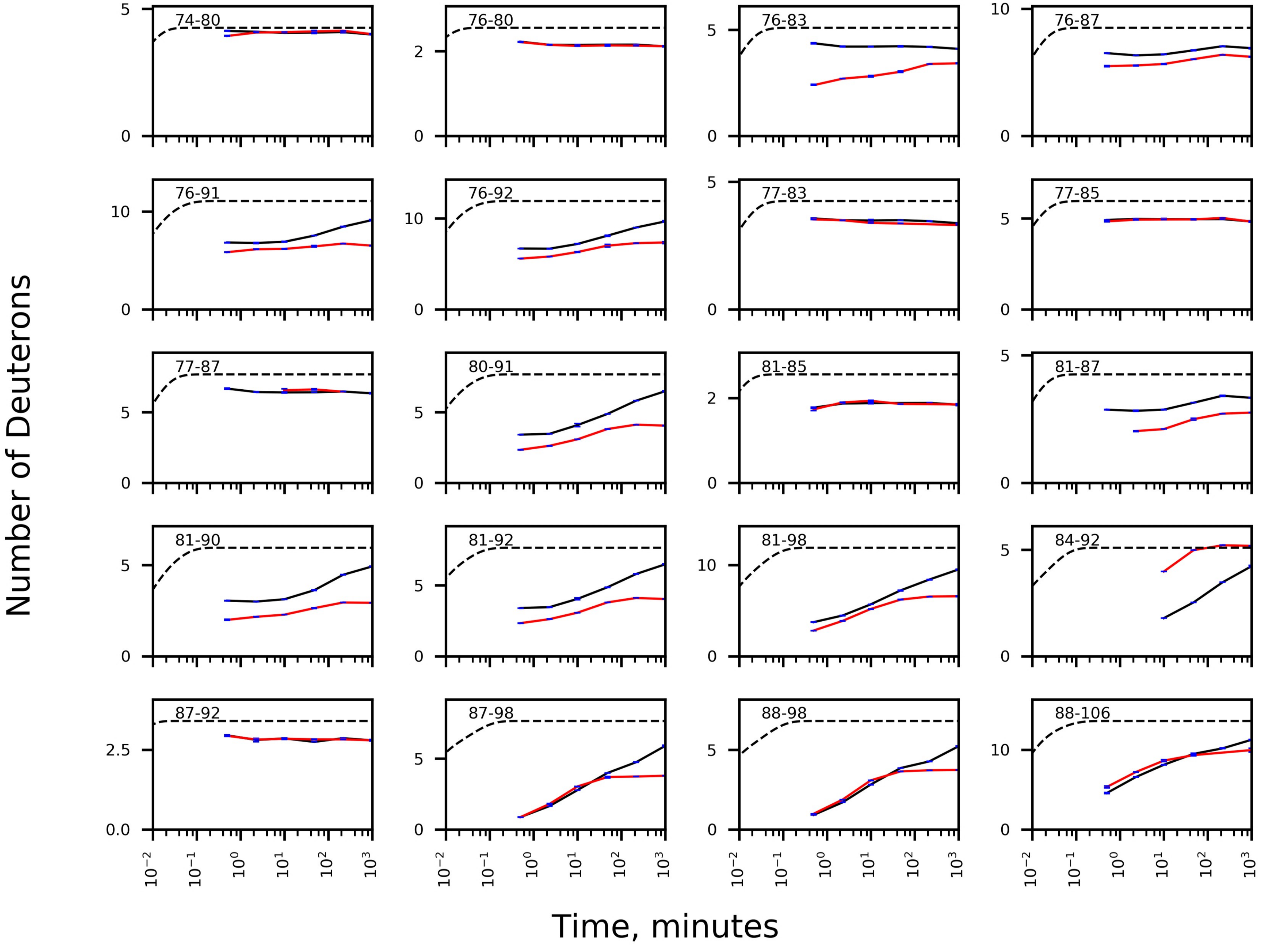

74-80
76-80
76-83
76-87
76-91
76-92
77-83
77-85
77-87
80-91
81-85
81-87
81-90
81-92
81-98
84-92
87-92
87-98
88-98
88-106
Number of Deuterons
Time, minutes

+E104v1

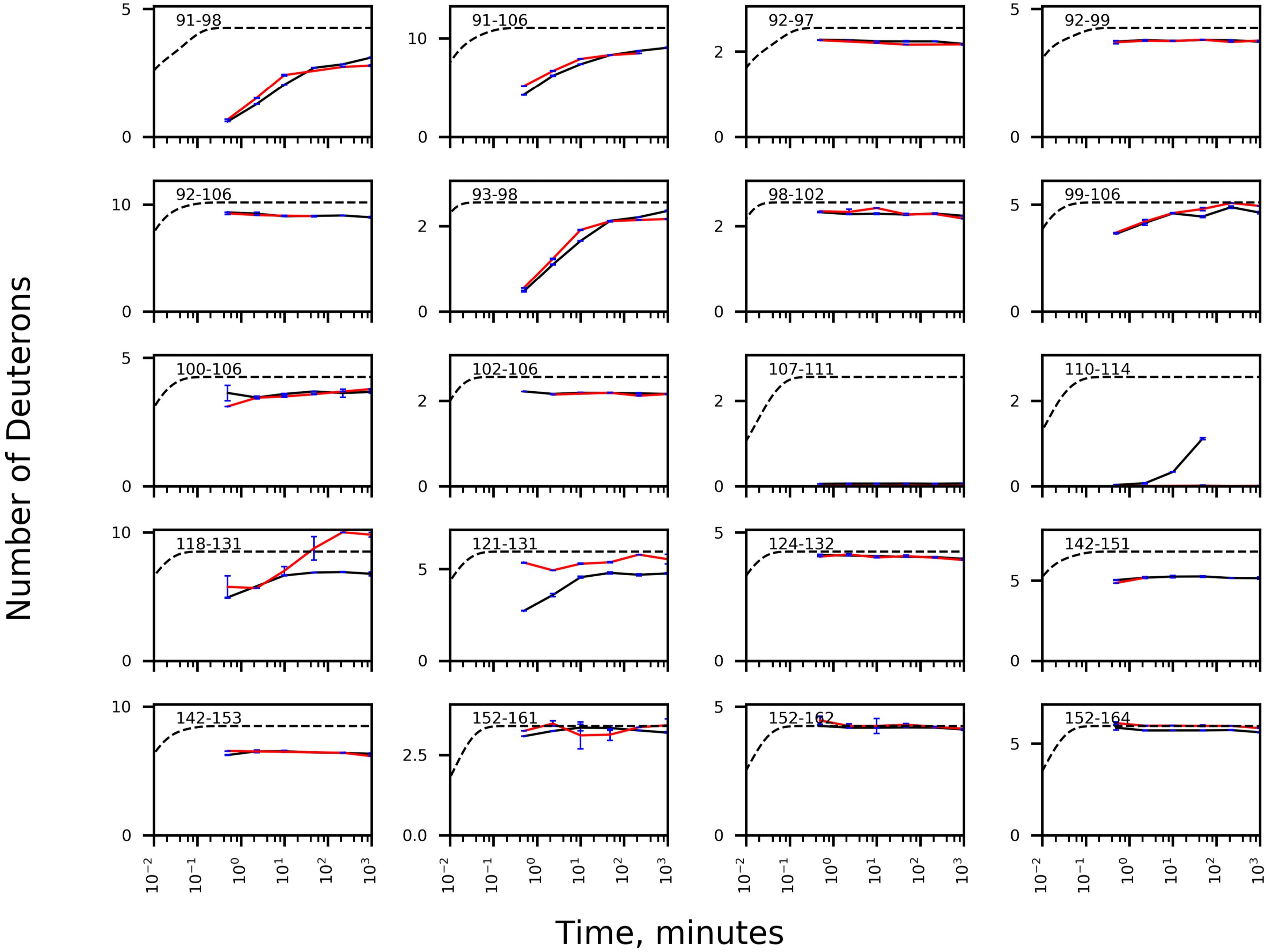

Number of Deuterons
Time, minutes
91-98
91-106
92-97
92-99
92-106
93-98
98-102
99-106
100-106
102-106
107-111
110-114
118-131
121-131
124-132
142-151
142-153
152-161
152-162
152-164

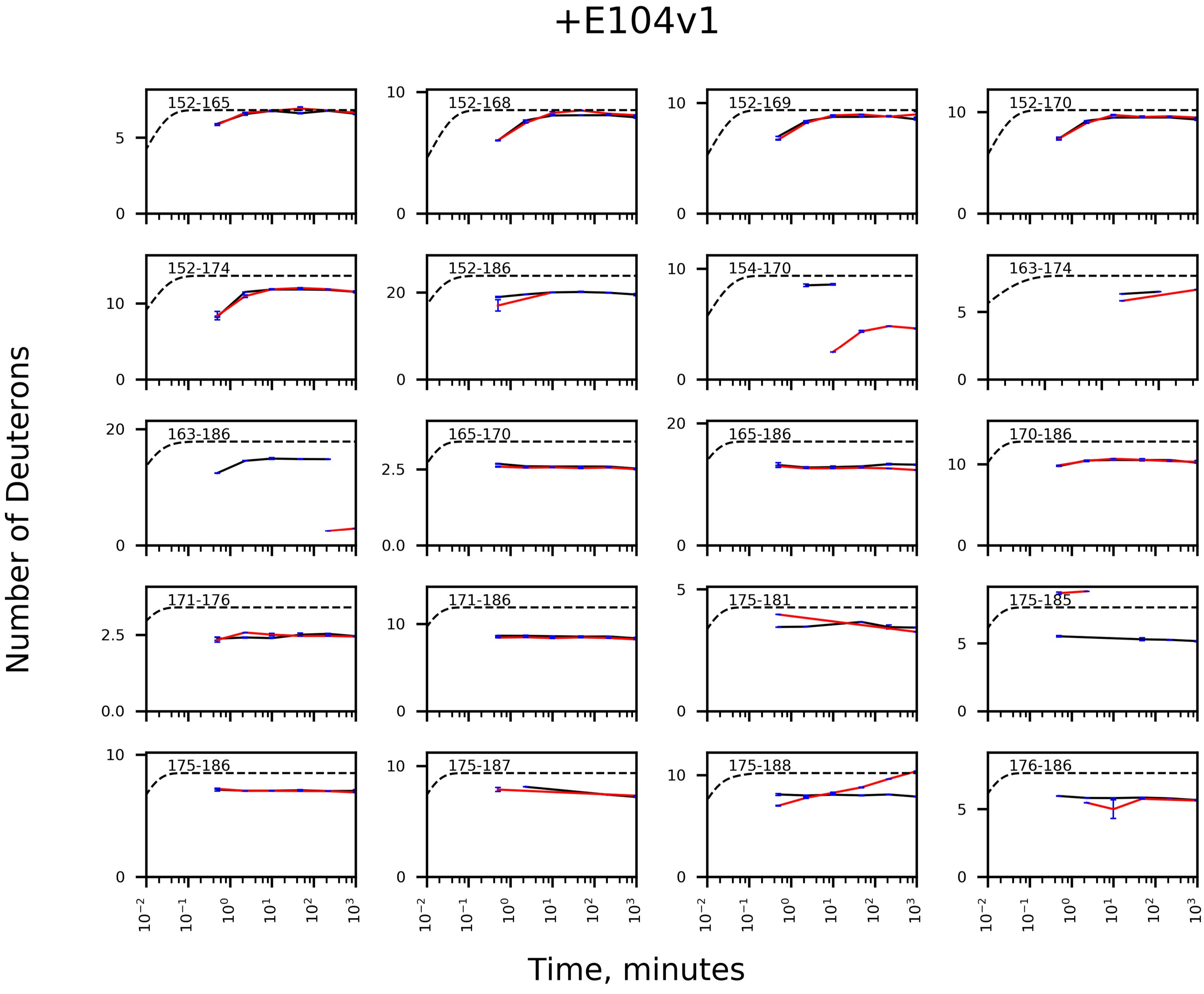
+E104v1
Number of Deuterons
Time, minutes
152-165
152-168
152-169
152-170
152-174
152-186
154-170
163-174
163-186
165-170
165-186
170-186
171-176
171-186
175-181
175-185
175-186
175-187
175-188
176-186

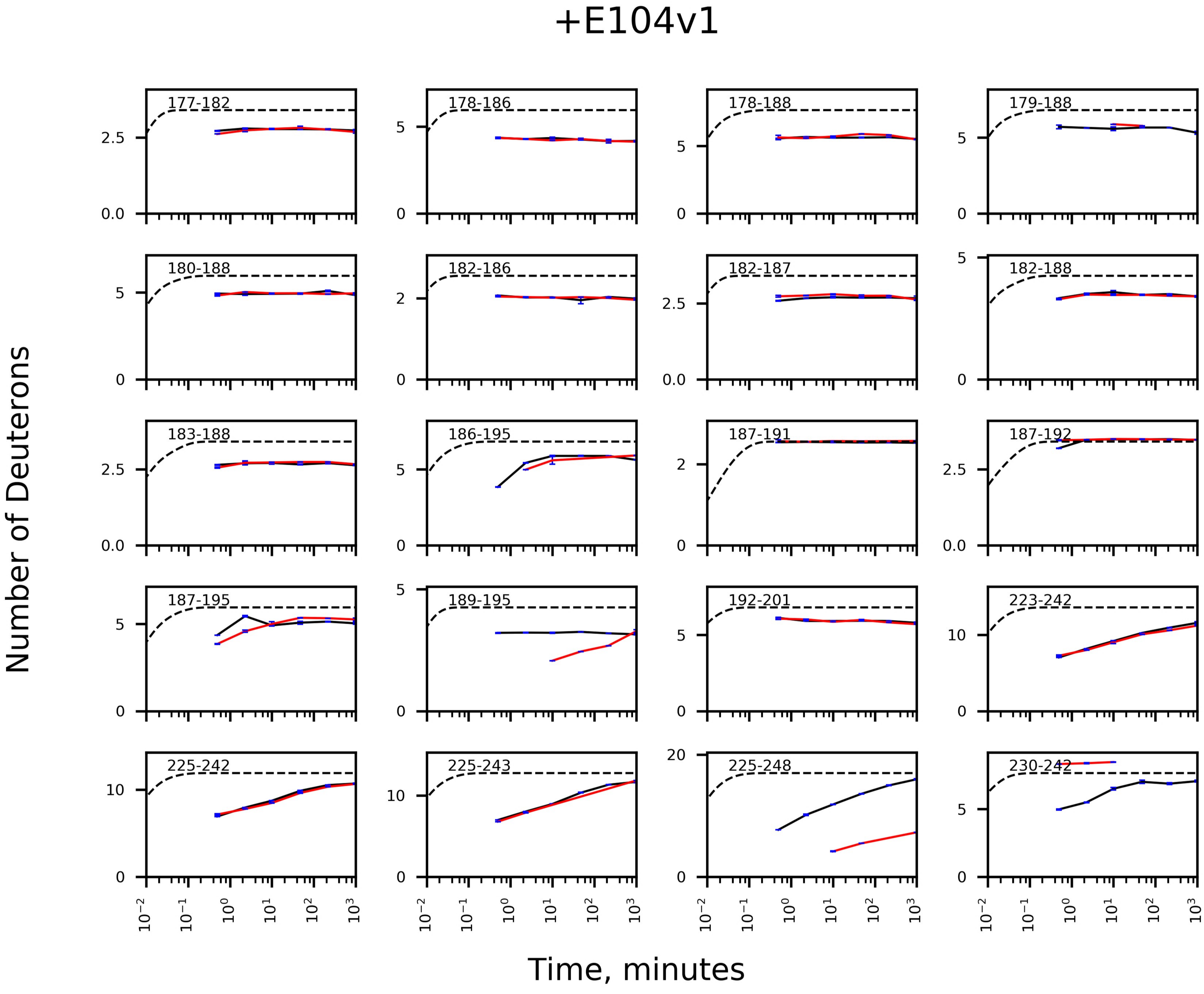

+E104v1
177-182
178-186
178-188
179-188
180-188
182-186
182-187
182-188
183-188
186-195
187-191
187-192
187-195
189-195
192-201
223-242
225-242
225-243
225-248
230-242
Number of Deuterons
Time, minutes

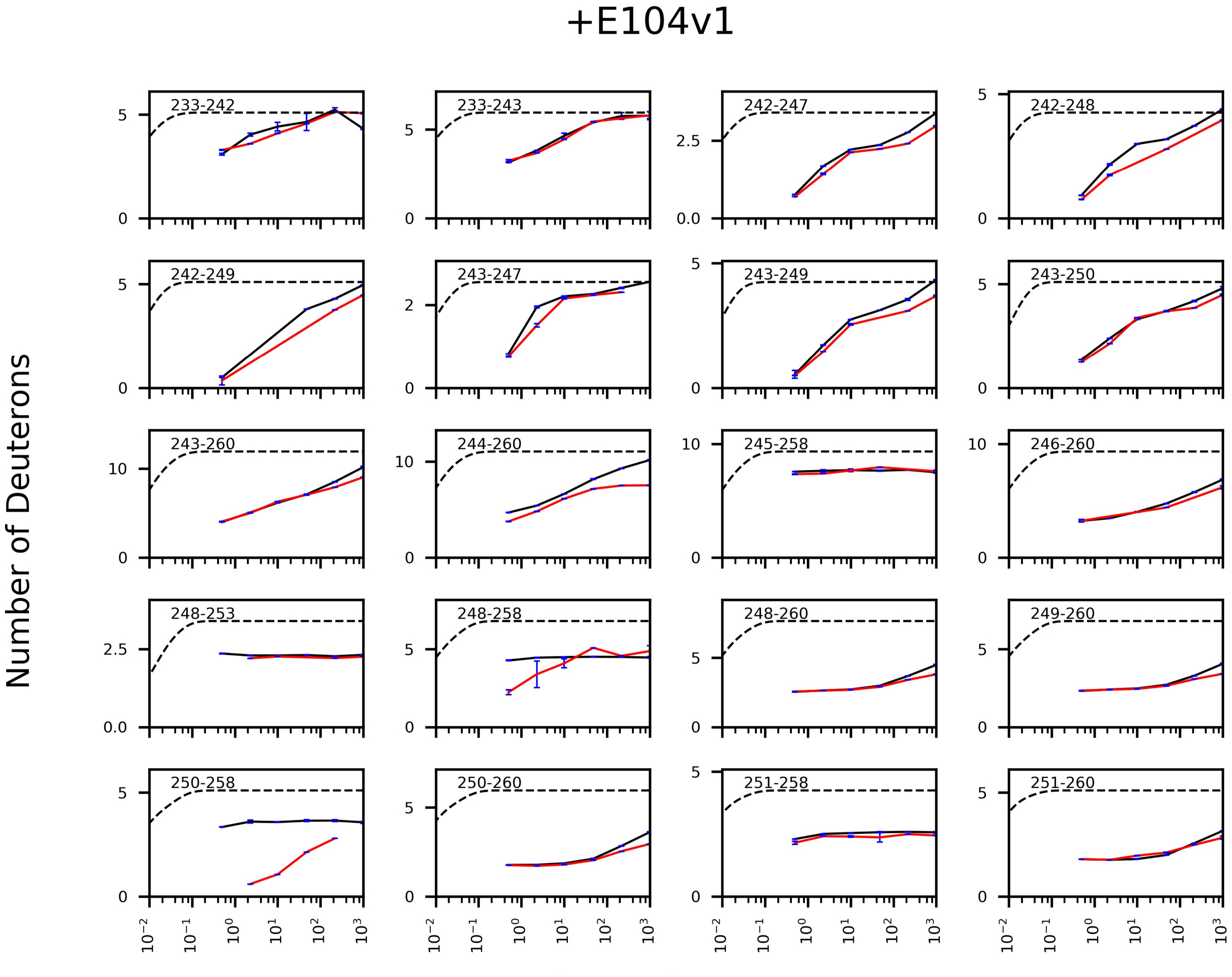
+E104v1
233-242
233-243
242-247
242-248
242-249
243-247
243-249
243-250
243-260
244-260
245-258
246-260
248-253
248-258
248-260
249-260
250-258
250-260
251-258
251-260
Number of Deuterons
Time, minutes

+E104v1

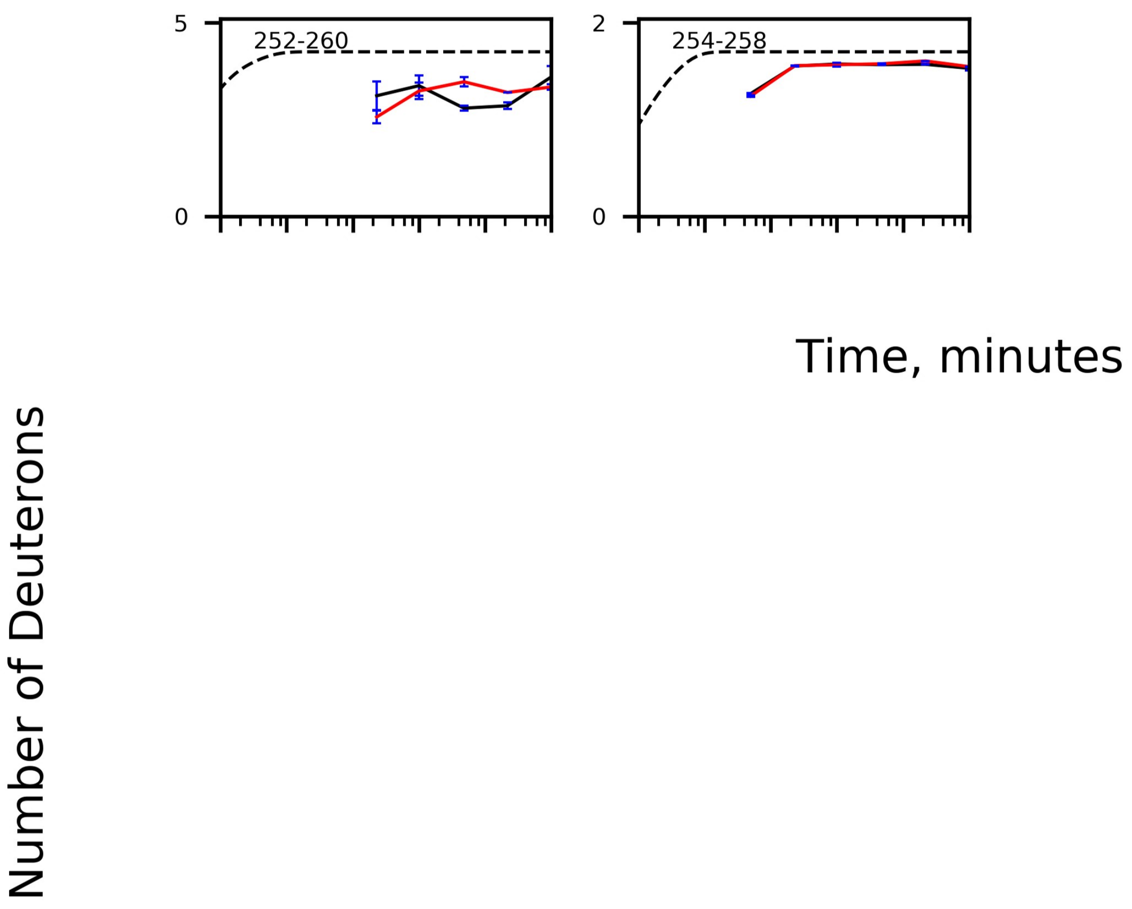

Supplementary Figure 10. HDX peptides of βI-tryptase in complex with E104.v2 Fab

+E104v2

Dashed black line is theoretical unprotected uptake trace. Solid black line is βI-tryptase alone. Solid red line is βI-tryptase in complex with E104.v2 Fab. Samples were analyzed in duplicate.

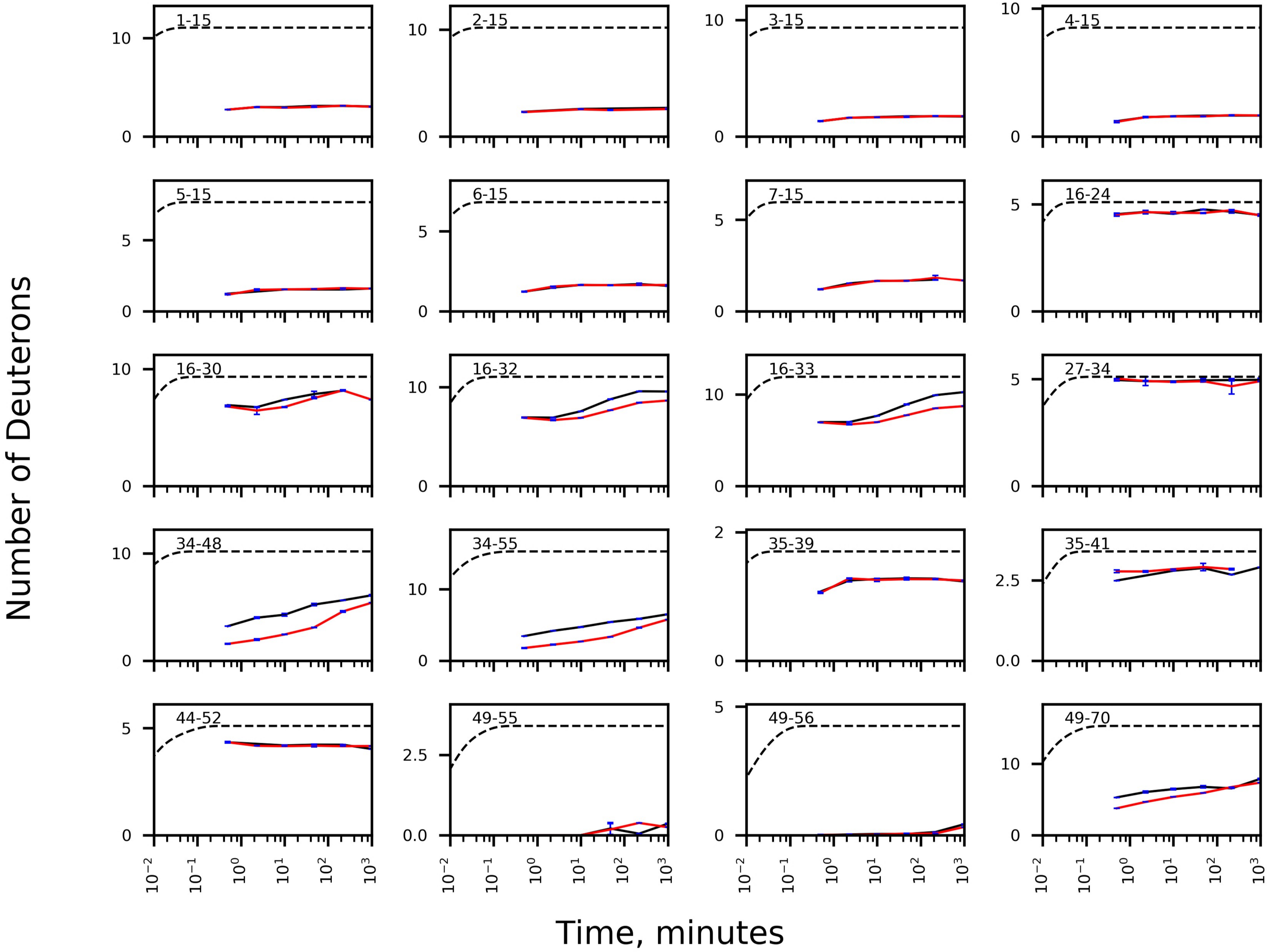

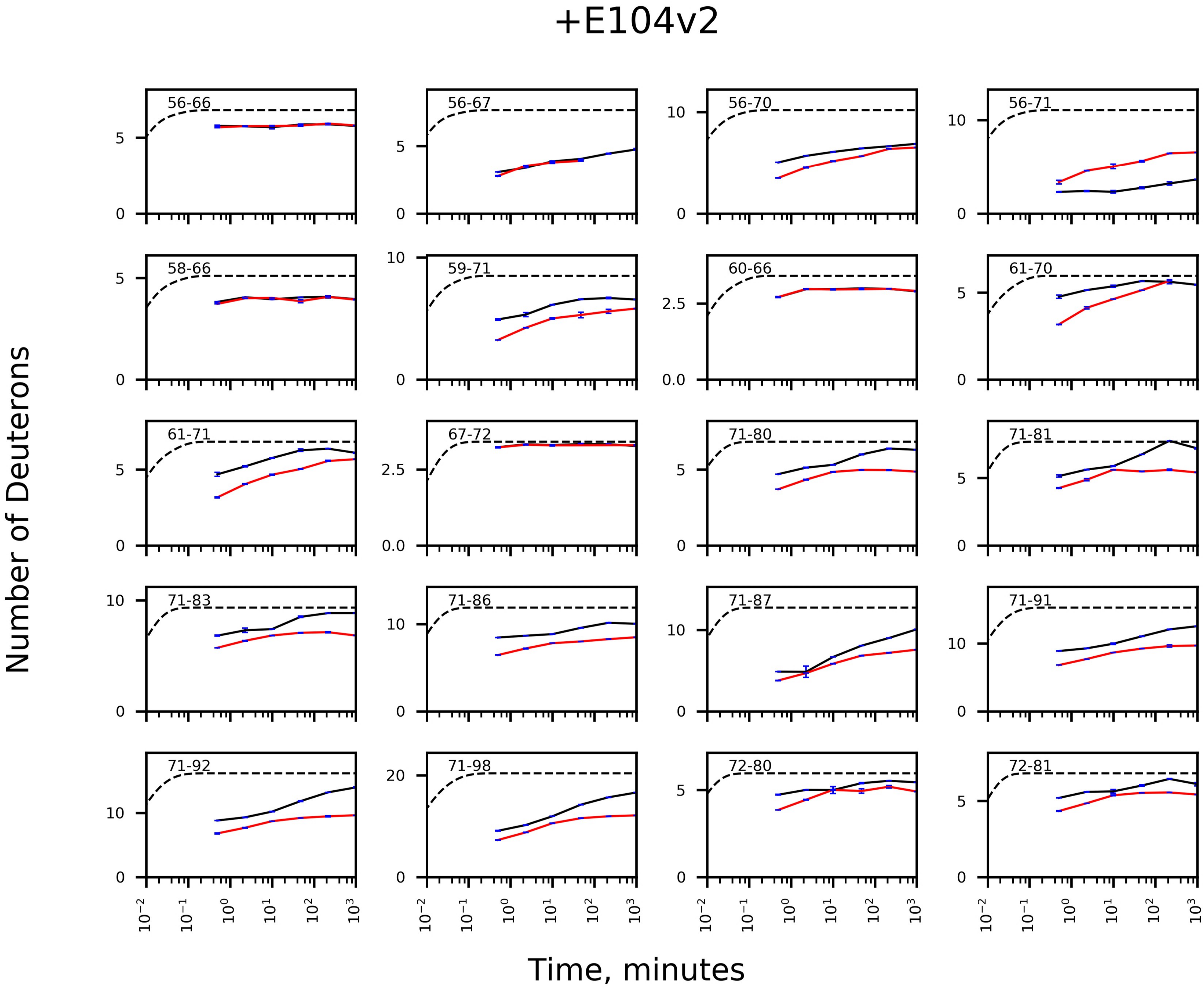
+E104v2
56-66
56-67
56-70
56-71
58-66
59-71
60-66
61-70
61-71
67-72
71-80
71-81
71-83
71-86
71-87
71-91
71-92
71-98
72-80
72-81
Number of Deuterons
Time, minutes

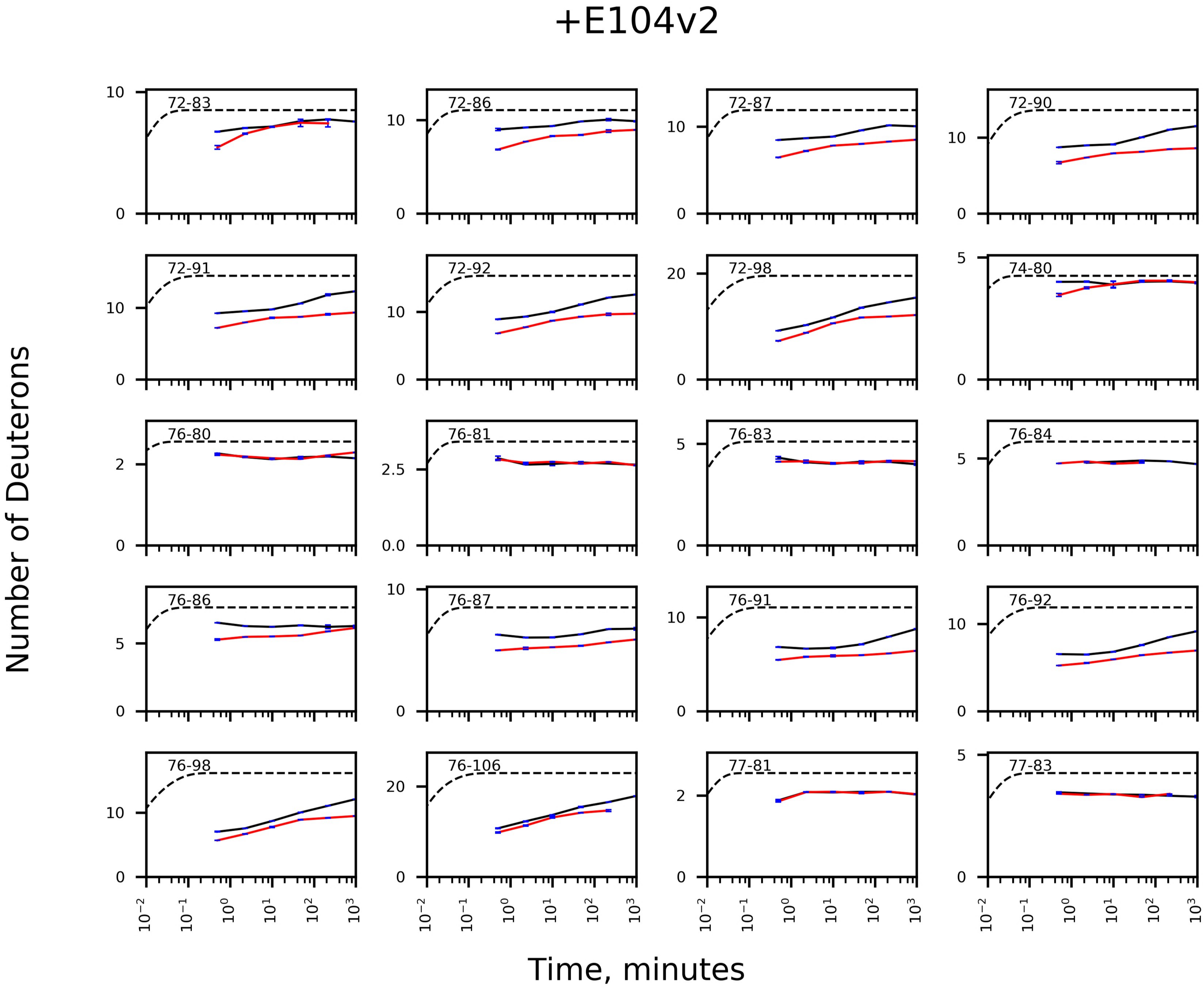
+E104v2
Number of Deuterons
Time, minutes
72-83
72-86
72-87
72-90
72-91
72-92
72-98
74-80
76-80
76-81
76-83
76-84
76-86
76-87
76-91
76-92
76-98
76-106
77-81
77-83

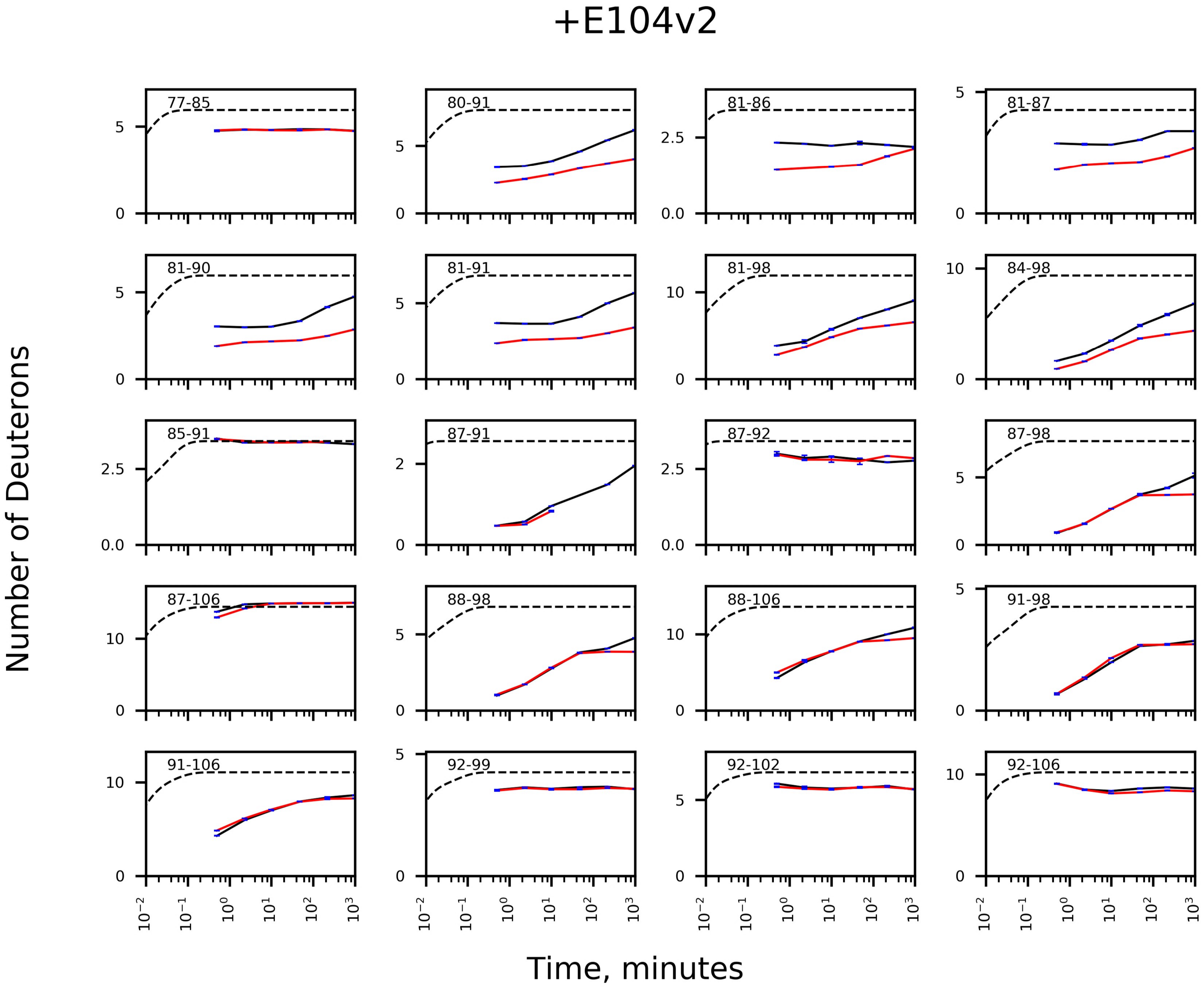

+E104v2
Number of Deuterons
Time, minutes
77-85
80-91
81-86
81-87
81-90
81-91
81-98
84-98
85-91
87-91
87-92
87-98
87-106
88-98
88-106
91-98
91-106
92-99
92-102
92-106

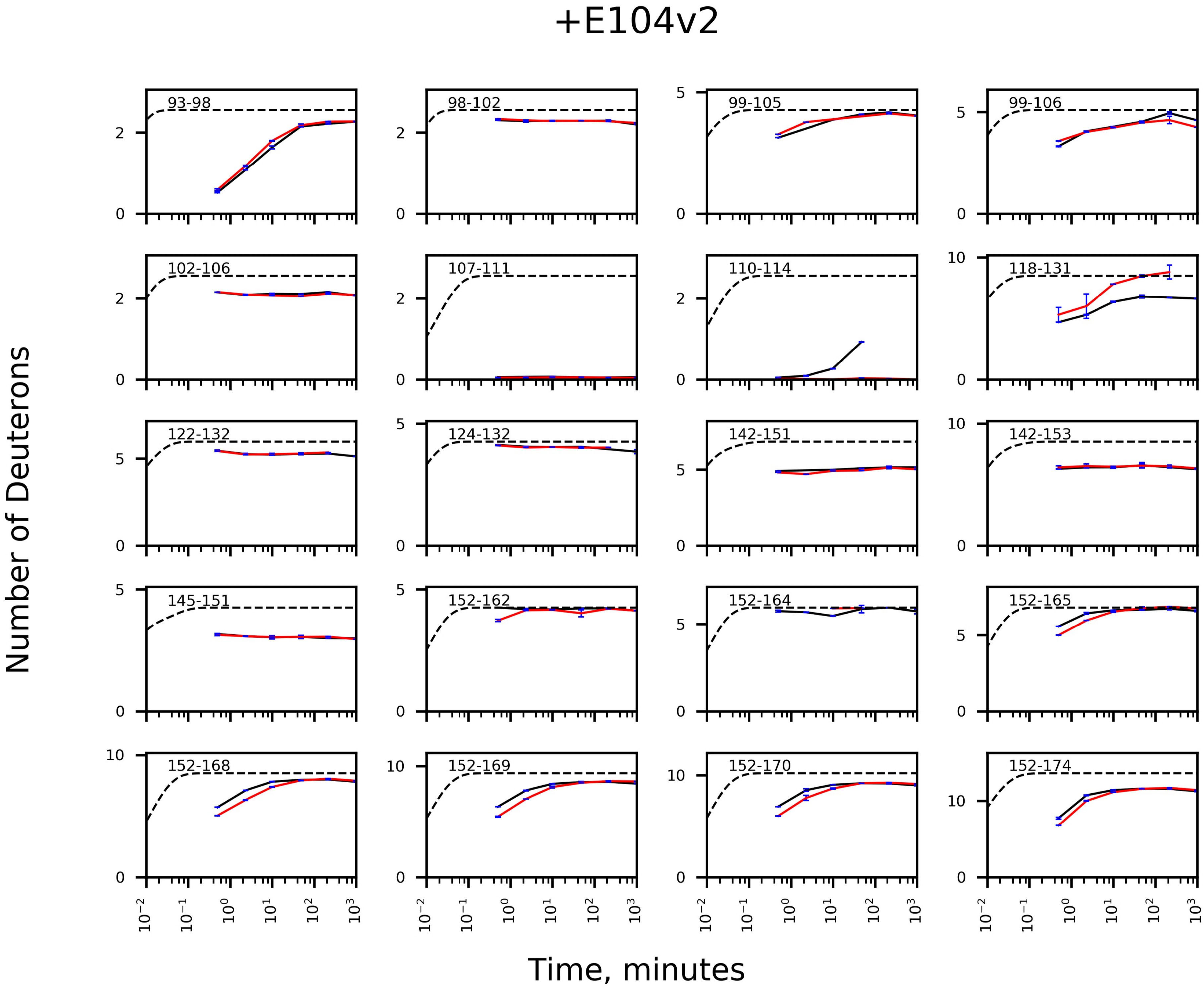

+E104v2
Number of Deuterons
Time, minutes
93-98
98-102
99-105
99-106
102-106
107-111
110-114
118-131
122-132
124-132
142-151
142-153
145-151
152-162
152-164
152-165
152-168
152-169
152-170
152-174

+E104v2

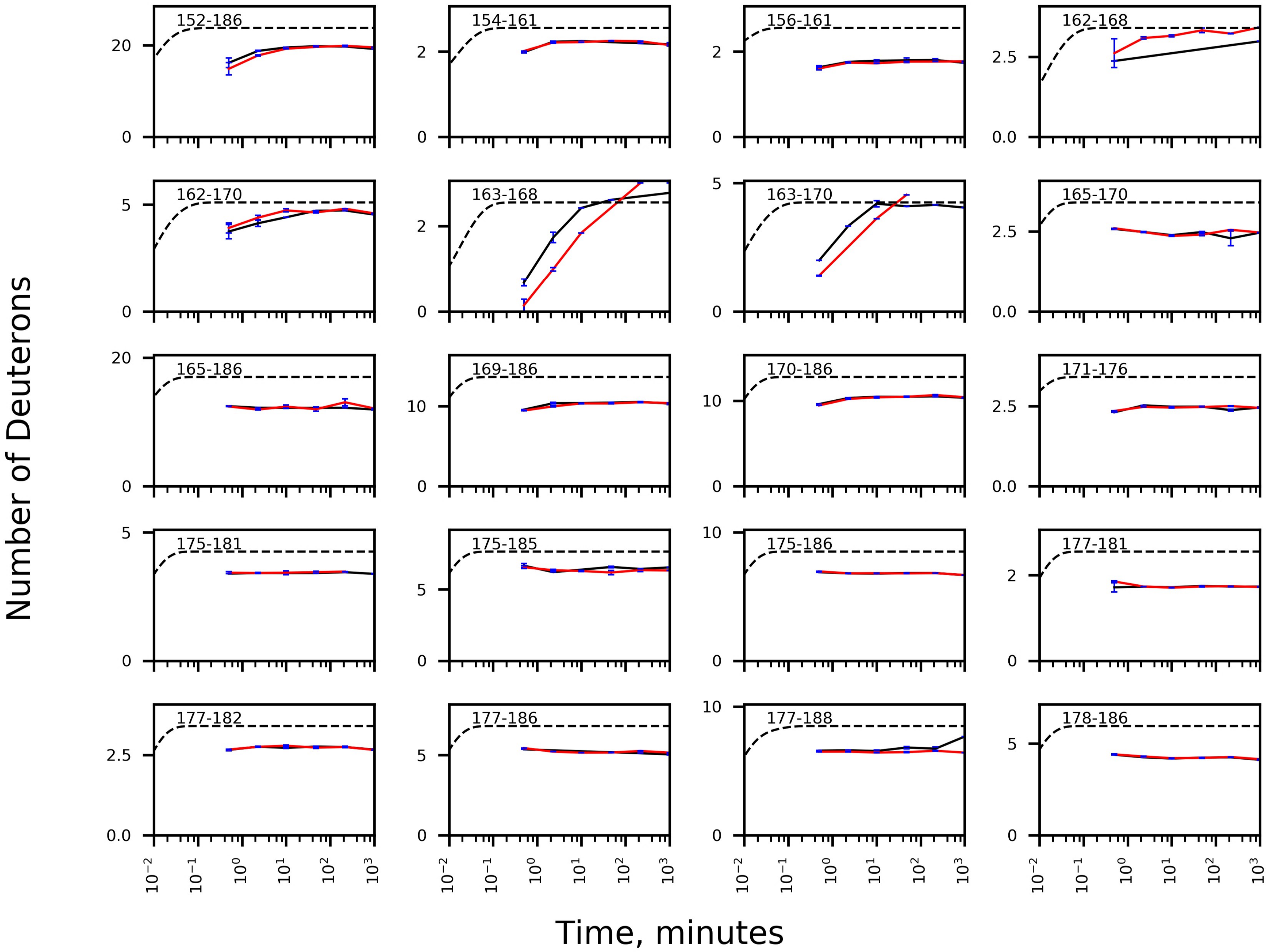

152-186
154-161
156-161
162-168
162-170
163-168
163-170
165-170
165-186
169-186
170-186
171-176
175-181
175-185
175-186
177-181
177-182
177-186
177-188
178-186
Number of Deuterons
Time, minutes

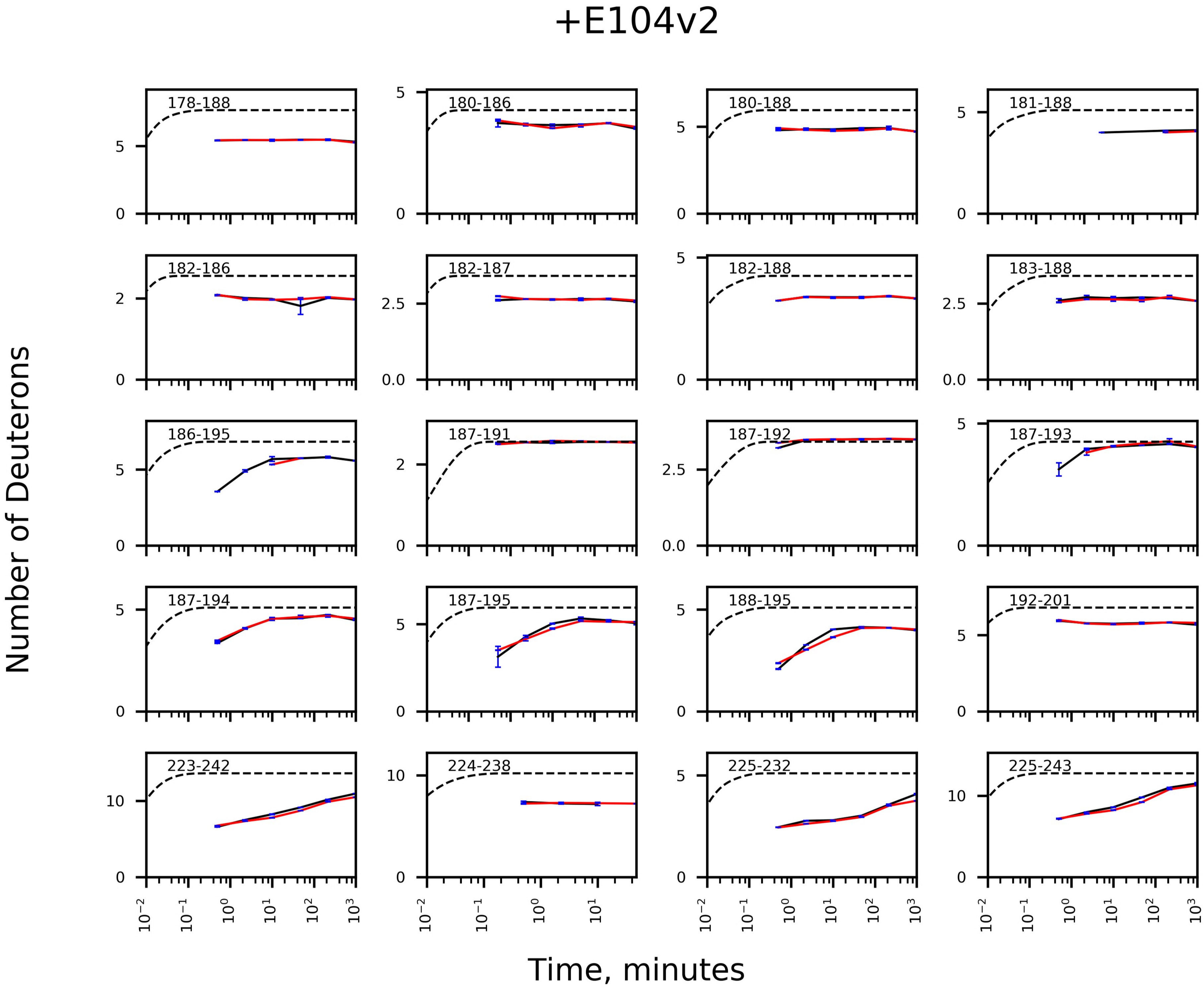

+E104v2
178-188
180-186
180-188
181-188
182-186
182-187
182-188
183-188
186-195
187-191
187-192
187-193
187-194
187-195
188-195
192-201
223-242
224-238
225-232
225-243
Number of Deuterons
Time, minutes

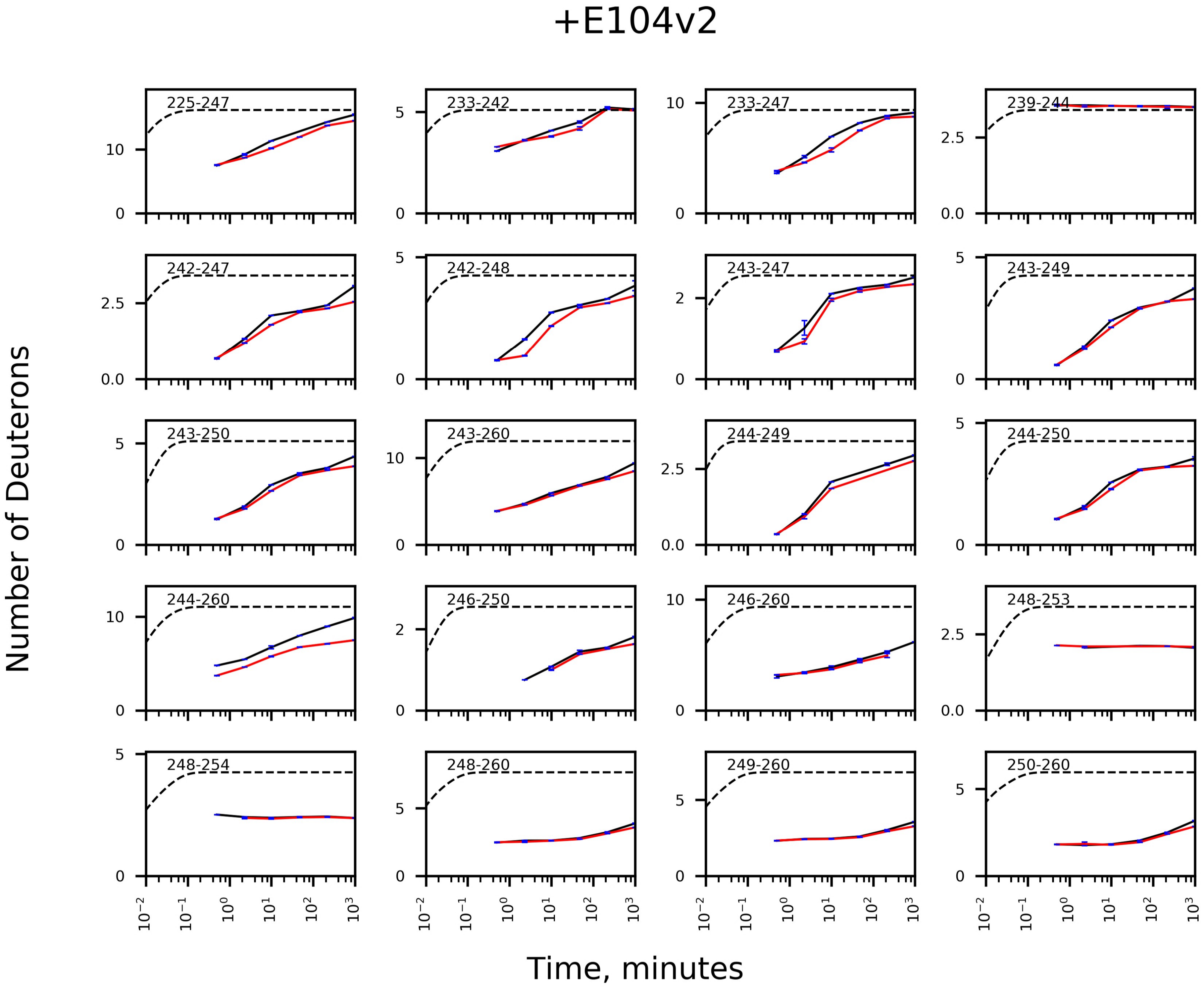

+E104v2
225-247
233-242
233-247
239-244
242-247
242-248
243-247
243-249
243-250
243-260
244-249
244-250
244-260
246-250
246-260
248-253
248-254
248-260
249-260
250-260
Number of Deuterons
Time, minutes

+E104v2

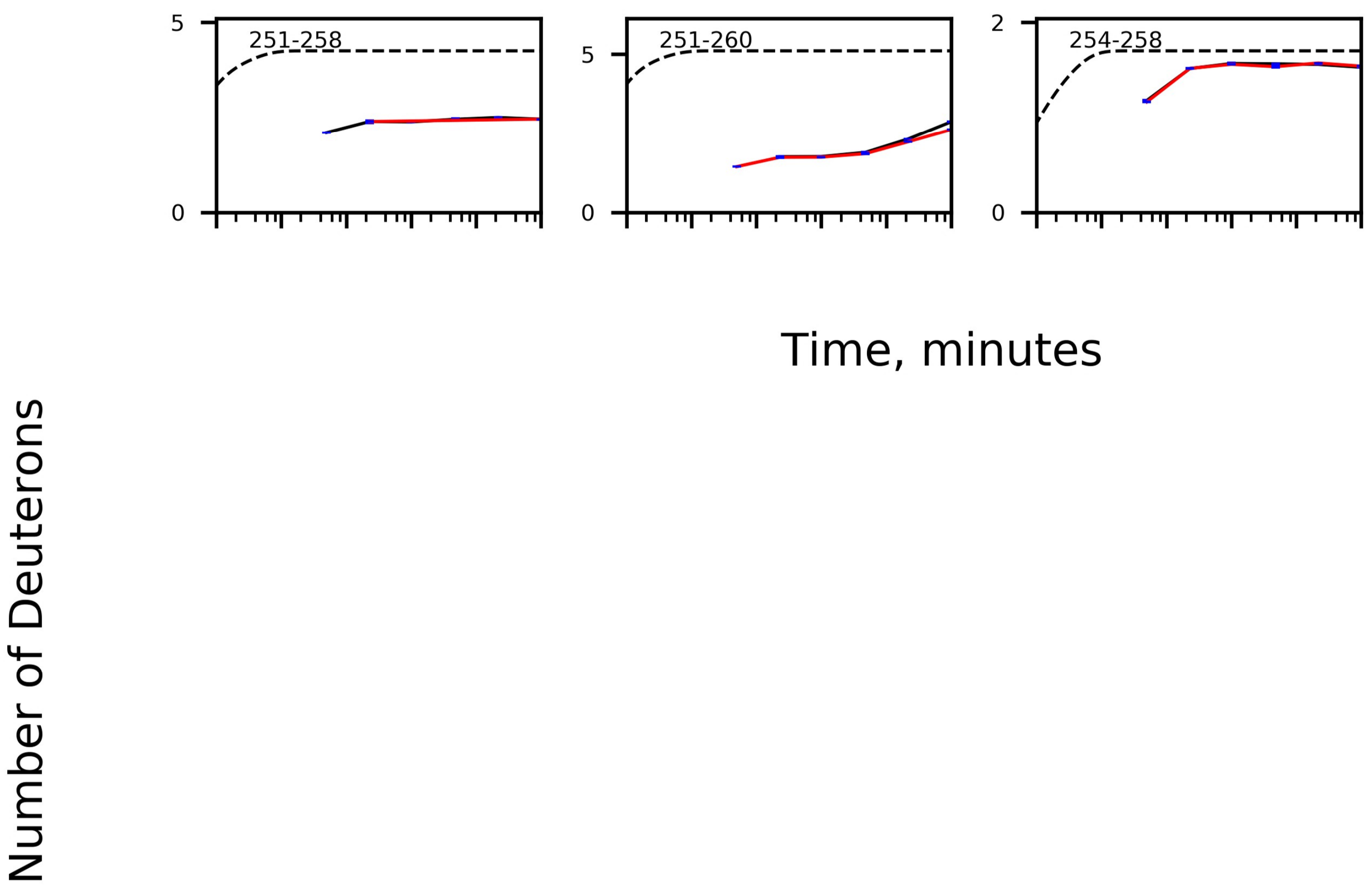
251-258
251-260
254-258
5
0
5
0
2
0
Time, minutes
Number of Deuterons

Supplementary Note 1

Significant differences in HX Experiments

The mean and range of data measured in duplicate are shown for each peptide included in the study for E104v1, Supplementary Fig. 9, and E104v2, Supplementary Fig. 10. A peptide was considered to report on a significant change in structural dynamics if the difference in mean deuterium incorporation was larger than the sum of the range in measurements for any particular time point. A heuristic filter was also included to eliminate the influence of false positives, requiring that any significant change should be confirmed by at least two overlapping peptides.

Interpretation of the HX results

HX experiments were the first in this series of work at Genentech to map the interactions of different Fabs with β1-tryptase providing a clue towards their mechanisms of inhibition. To maximize the utility of this data, peptides showing significant difference in deuterium uptake were compared with one another manually to refine the labeling pattern, the results are shown in Supplementary table 2 and are mapped onto the structure in Fig. 2f. Those results suggested that E104.v2 augmented interactions in β1-tryptase critical for stabilization of the tetramer, unlike E104.v1. The striking similarity between crystallographic contacts determined later and those extracted from HX experiments reinforces the effectiveness of manual analysis of experimental results. Orthogonal experiments included in the main text provided additional confidence in the manual interpretation, but a more rigorous statistical approach to defining significant changes between datasets is also desirable.

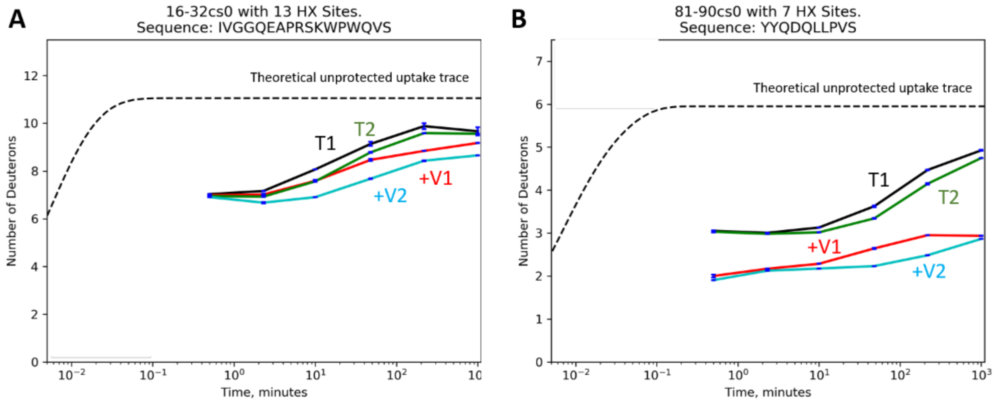

.

Supplementary Figure 11: Two representative peptides covering the two regions of β1-tryptase where binding to E104.v1 (represented by "V1") caused a much less pronounced effect than binding to E104.v2 (represented by "V2") as determined by the differential analysis of protection factors. Traces labeled T1 and T2 refer to the tryptase-only uptake traces that were collected as matched controls for extracting protection factors for V1 and V2 bound states, respectively. This notation is also used in supplemental text. Bars that are shown on the traces represent the range of duplicate measurements.

The experimental data between E104.v1 and v2 could not be directly compared without correcting for potential systematic errors because experiments were conducted at different times

and chemical exchange rates are sensitive to differences in environmental variables. This is not a problem in matched experiments due to randomization (timepoints and replicates are scrambled, the longest timepoint is penultimate, the shortest timepoint is last, replicates and all other timepoints are randomized completely). As a consequence, systematic error would manifest as larger error bars increasing the significance threshold. Since unbound measurements read the same information in both datasets (and should be coincident sans systematic error), the difference in rates between experiments can be computed as previously described using an empirical protection factor[1] (PF). The computed PF between bound measurements in separate experiments can be corrected using the change in PF between apo traces in those experiments. A method to compute an uncertainty estimate in empirical protection factors is introduced below. To briefly summarize before describing the method, two regions of β1-tryptase are identified to be significantly more retarded by the binding of v2. Representative peptides are shown in Supplementary Figure 11. These two regions were generally consistent with the nuanced manual analysis described above and we defaulted to the higher resolution and orthogonally corroborated manual description of HX results in the main text.

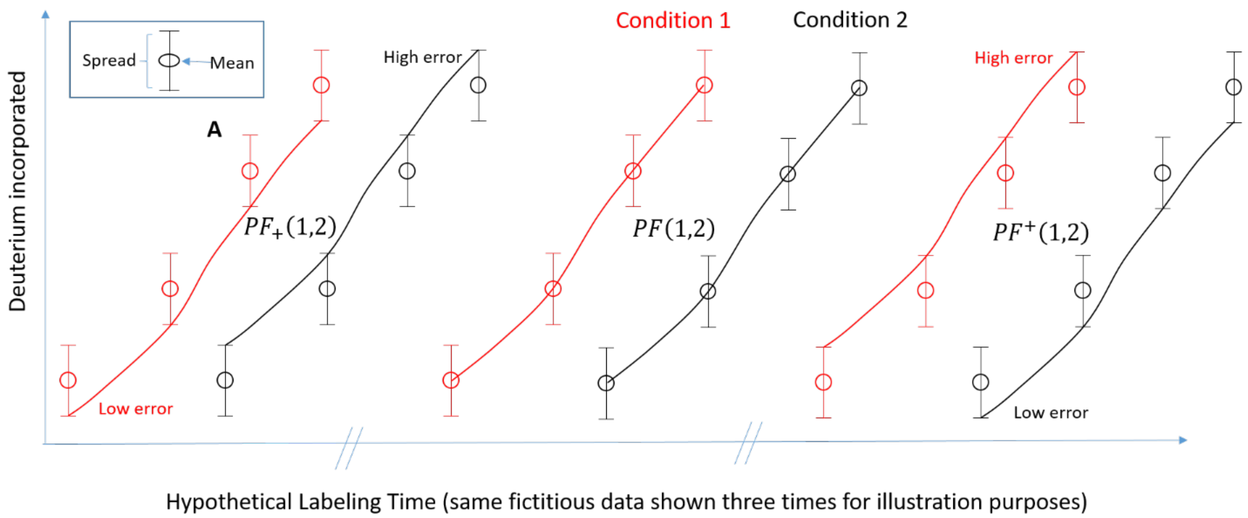

Supplementary figure 12. As originally described, an empirical protection factor can be extracted for any two HX MS traces (red/black lines above) provided that there is enough overlap in deuterium uptake[1]. Here three different sets of update traces are constructed using either the mean or boundaries of hypothetical data. Using the mean value of replicates for both sets (middle) produces the empirical PF as described in the original manuscript, ($PF(1,2)$). PF boundaries are then produced in the same fashion but by using traces constructed from the boundaries of measurements instead of their mean, as is shown to the right and left of center, $PF^+(1,2)$, and $PF_+(1,2)$, respectively. Assignment is arbitrary as long as the the pattern of using the high bound from a condition with the low bound of the other condition for one boundary is opposite of the other, as is suggested by the diagram. These error bars are hypothetical, and represent general variability in measurements.

Uncertainty in Empirical Protection Factors

An empirical protection factor, $PF(1,2)$, describes a coefficient that maps the changes in exchange rates from condition 2 onto condition 1. Instructions on how to determine this value are described in the original publication[1]. Working in log space simplifies the calculations. The uncertainty or error in this work is represented as:

$$\boldsymbol{s} = \{\log PF^{+}(1,2), \log PF_{+}(1,2)\} \quad (1)$$

$$\delta \log PF(1,2) = \max \boldsymbol{s} - \min \boldsymbol{s} \quad (2)$$

The assignment of boundaries, $PF^{+}(1,2)$ or $PF_{+}(1,2)$, shown in Supplementary figure 12, is arbitrary due to the max/min operators as long as the pattern of comparing the high error construct from one condition with the low error construct for one boundary is the opposite of the other boundary. This procedure endows the computed protection factor with uncertainty: $\log PF(1,2) \pm \delta \log PF(1,2)$.

Extending this for use in correcting the bound data with apo information, an arbitrary value, is introduced to represent the corrected value $\frac{PF(v1,v2)}{PF(t1,t2)}$, in log space and the propagated uncertainty:

$$\mathrm{R} = \log PF(v1, v2) - \log PF(t1, t2) \quad (3)$$

$$\delta R = \sqrt{\delta \log PF(v1, v2)^2 + \delta \log PF(t1, t2)^2} \quad (4)$$

where v1 and v2 represent β1-tryptase bound to either E104v1 or E104v2, and t1 or t2 represent the contemporaneous measurement of unbound β1-tryptase, respectively, for each experiment. The corrected measurement, with uncertainty, is $R \pm \delta R$.

Significant differences are defined as those where $|R| > \delta R$ and $\frac{PF(v1,v2)}{PF(t1,t2)}$ is either >2 or < 0.5. The second requirement focuses this analysis on regions with more substantial energetic differences but it is not statistically necessary. Similar to the individual analyses described earlier, significant findings are corroborated by at least one overlapping peptide. A summary of the HDX data is included as Supplementary Data 1 and all calculations are included in an attached Supplementary Data 2. Fewer peptides are amenable to this analysis because the extraction of an empirical protection factor requires certain qualities in the data to be present as described[1], most importantly is the overlap in uptake. For this analysis, the required overlap between traces in deuterium uptake was set to 0.05*N, where N equals the number of exchange sites, and only peptides where two protection factors could be computed were included in the analysis**.** Therefore, the resolution is much lower in this more rigorous analysis.

Supplementary References

1. Walters, B.T. Empirical Method To Accurately Determine Peptide-Averaged Protection Factors from Hydrogen Exchange MS Data. *Anal Chem* **89**, 1049-1053 (2017).
