# Supplementary material for: Bivalent antibody pliers inhibit β-tryptase by an allosteric mechanism dependent on the IgG hinge
Source: Nat Commun. 2020 Dec 22;11:6435. doi: 10.1038/s41467-020-20143-x (PMC7755903; doi:10.1038/s41467-020-20143-x)
Supplement: Supplementary file 3 — Description of Additional Supplementary Files [file 41467_2020_20143_MOESM3_ESM.docx]

Description of Additional Supplementary Files

File Name: Supplementary Data 1

Description: Summary table of HDX data and analysis

File Name: Supplementary Data 2

Description: Significant calculations for all of the peptides analyzed in HDX data
