# Supplementary material for: Bivalent antibody pliers inhibit β-tryptase by an allosteric mechanism dependent on the IgG hinge
Source: Nat Commun. 2020 Dec 22;11:6435. doi: 10.1038/s41467-020-20143-x (PMC7755903; doi:10.1038/s41467-020-20143-x)
Supplement: Supplementary file 6 — Reporting Summary [file 41467_2020_20143_MOESM6_ESM.pdf]

nature research

Corresponding author(s): James Koerber, Bob Lazarus

Last updated by author(s): 10/27/2020

Reporting Summary

Nature Research wishes to improve the reproducibility of the work that we publish. This form provides structure for consistency and transparency in reporting. For further information on Nature Research policies, see Authors & Referees and the Editorial Policy Checklist .

Statistics

For all statistical analyses, confirm that the following items are present in the figure legend, table legend, main text, or Methods section.

n/a	Confirmed	
☐	☒	The exact sample size (n) for each experimental group/condition, given as a discrete number and unit of measurement
☐	☒	A statement on whether measurements were taken from distinct samples or whether the same sample was measured repeatedly
☒	☐	The statistical test(s) used AND whether they are one- or two-sided<br>*Only common tests should be described solely by name; describe more complex techniques in the Methods section.*
☒	☐	A description of all covariates tested
☒	☐	A description of any assumptions or corrections, such as tests of normality and adjustment for multiple comparisons
☐	☒	A full description of the statistical parameters including central tendency (e.g. means) or other basic estimates (e.g. regression coefficient) AND variation (e.g. standard deviation) or associated estimates of uncertainty (e.g. confidence intervals)
☒	☐	For null hypothesis testing, the test statistic (e.g. F, t, r) with confidence intervals, effect sizes, degrees of freedom and P value noted<br>*Give P values as exact values whenever suitable.*
☒	☐	For Bayesian analysis, information on the choice of priors and Markov chain Monte Carlo settings
☒	☐	For hierarchical and complex designs, identification of the appropriate level for tests and full reporting of outcomes
☒	☐	Estimates of effect sizes (e.g. Cohen's d, Pearson's r), indicating how they were calculated

Our web collection on statistics for biologists contains articles on many of the points above.

Software and code

Policy information about availability of computer code

Data collection	XDS
Data analysis	Eman2 e2boxer v2.1, IMAGIC v10, Relion v3, pdb2mrc in Eman v2.1, Chimera v1.12, Coot, REFMAC5, Phenix.refine, ExMS v1, MSConvertGui v3.0.5741, Mascot v2.1, Buster v2.11.2, KaleidaGraph v4.1

For manuscripts utilizing custom algorithms or software that are central to the research but not yet described in published literature, software must be made available to editors/reviewers. We strongly encourage code deposition in a community repository (e.g. GitHub). See the Nature Research guidelines for submitting code & software for further information.

Data

Policy information about availability of data

All manuscripts must include a data availability statement. This statement should provide the following information, where applicable:

- Accession codes, unique identifiers, or web links for publicly available datasets
- A list of figures that have associated raw data
- A description of any restrictions on data availability

An electron microscopy density map for the negative staining reconstruction of βI-tryptase tetramer bound to E104.v1 Fab has been deposited in the Electron Microscopy Data Bank (https://www.ebi.ac.uk/pdbe/emdb/) under accession code EMD-21389. The crystal structure of the βI-tryptase tetramer bound to E104.v1 Fab has been deposited in the protein databank (PDB) with PDB accession code 6VVU.

Field-specific reporting

Please select the one below that is the best fit for your research. If you are not sure, read the appropriate sections before making your selection.

Life sciences study design

All studies must disclose on these points even when the disclosure is negative.

Sample size	Three to four biological replicates were performed, which was sufficient to show data reproducibility. No sample-size calculations were performed.
Data exclusions	No data was excluded from analysis.
Replication	Three or four biological replicates were used for cell-based assays and in vitro enzymatic assays. Similar results were obtained in each replicate.
Randomization	Randomization was not needed for this study, since samples were not divided into groups.
Blinding	No blinding was performed since group analysis was not performed.

Reporting for specific materials, systems and methods

We require information from authors about some types of materials, experimental systems and methods used in many studies. Here, indicate whether each material, system or method listed is relevant to your study. If you are not sure if a list item applies to your research, read the appropriate section before selecting a response.

Materials & experimental systems

n/a	Involved in the study	
☐	☒	Antibodies
☐	☒	Eukaryotic cell lines
☒	☐	Palaeontology
☒	☐	Animals and other organisms
☒	☐	Human research participants
☒	☐	Clinical data

Methods

n/a	Involved in the study	
☒	☐	ChIP-seq
☒	☐	Flow cytometry
☒	☐	MRI-based neuroimaging

Antibodies

Antibodies used	Mouse anti-human IgG (Human antibody capture kit; GE Healthcare #BR100839)<br>E104 (generated by Genentech and described in this paper)
Validation	E104 validation was confirmed by SPR and crystal structure as described by this manuscript.<br>According to GE Healthcare product website, Anti-Human IgG (Fc) consists of a monoclonal mouse anti-human IgG (Fc) antibody of IgG1 isotype, prepared by affinity chromatography on Protein A.

Eukaryotic cell lines

Policy information about cell lines

Cell line source(s)	Human bronchial smooth muscle cells (Lonza), Expi293F (ThermoFisher Scientific)
Authentication	None of the cell lines used were authenticated.
Mycoplasma contamination	Negative
Commonly misidentified lines (See ICLAC register)	No commonly misidentified cell lines were used.
